# Tackling vascular wilt disease: A signaling cascade to strengthen the plant cell wall

**DOI:** 10.1093/plcell/koae299

**Published:** 2024-11-18

**Authors:** Shanice S Webster

**Affiliations:** Assistant Features Editor, The Plant Cell, American Society of Plant Biologists; Howard Hughes Medical Institute, Chevy Chase, MD 20815, USA; Department of Biology, Duke University, Durham, NC 27708, USA

A critical challenge to agricultural productivity and human health is the devastating impact of biotic stresses. Among these, vascular wilt disease has emerged as a particularly severe category, affecting an extensive range of more than 200 plant species globally (Mansfield et al. 2012). The disease is caused by pathogens and pests that proliferate in the plant xylem and block transport of water and nutrients to leaves, causing systemic wilting. As a defense mechanism, plants increase lignin deposition in the xylem vessels, forming a physical barrier to prevent pathogen spread and potentially impede the delivery of effectors into plant cells. In recent work, **Bingqian Wang and colleagues** (Wang et al. 2024) elucidate a mechanism underlying this defense mechanism, demonstrating the importance of RD26 in increasing xylem lignification and a key regulatory role of the receptor kinase FERONIA in suppressing pathogen infection and in modulating the growth-defense tradeoff during this process.

Elevated lignin levels during infection correlate with enhanced resistance to vascular pathogens (Ferreira et al. 2017; Novo et al. 2017). However, the key immune signaling components and pathways that link pathogen reception with cell wall lignification are largely unknown. Using fluorescent microscopy analyses, Wang et al. confirmed that the vascular pathogen *Ralstonia solanacearum* successfully colonizes the root xylem of Arabidopsis (*A. thaliana*), where it elicits increased lignin production, accompanied by significant upregulation of the lignin biosynthesis gene PAL1. These studies establish cell wall lignification as a critical defense strategy against pathogen invasion in the xylem (Fig.).

**Figure.**
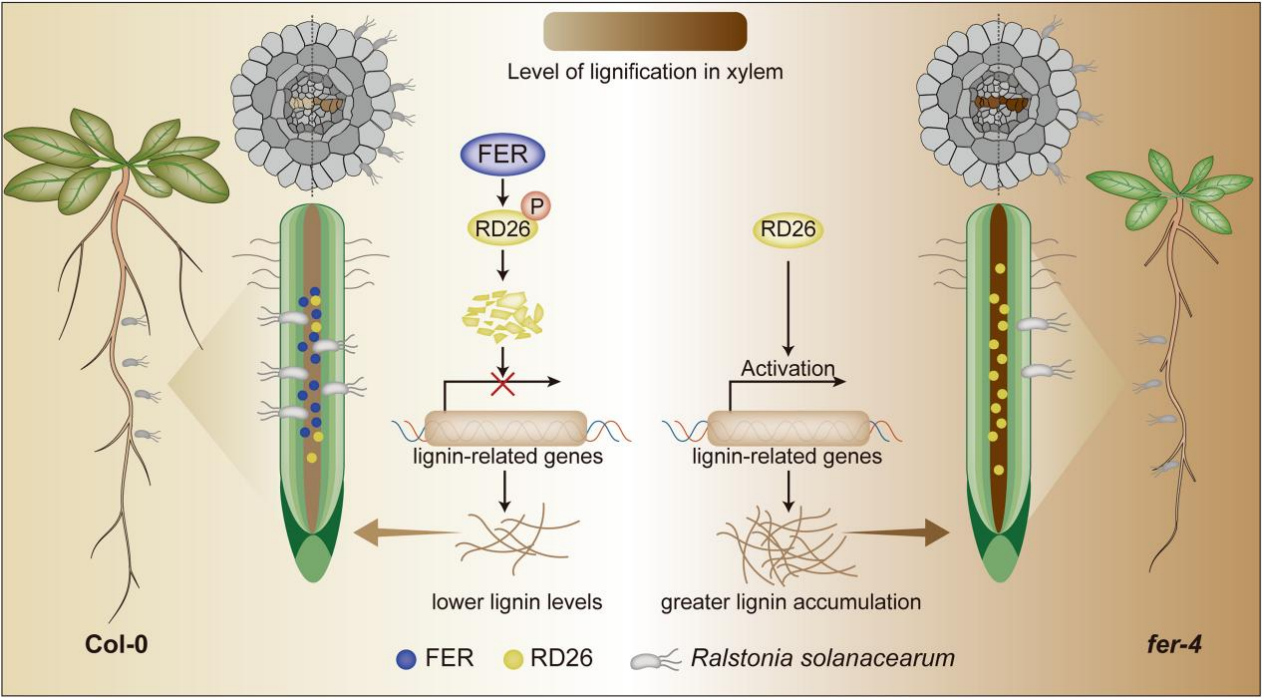
Model of how FER-RD26 signaling mechanism regulates cell wall lignification to increase plant resistance against the vascular pathogen, *Ralstonia solanacearum*. Reprinted from Wang et al. (2024), Figure 9.

Previous work established FERONIA (FER) as a crucial regulator of cell wall integrity (Feng et al. 2018), with recent findings indicating its role in cell wall modification and *R. solanacearum* resistance (Liu et al. 2023; Liu et al. 2023). Using a *fer* mutant, Wang et al. revealed that *fer* has higher lignin levels and decreased pathogen colonization compared with wild-type plants, suggesting that FER is a negative regulator of lignin deposition and, consequently, of *R. solanacearum* resistance.

Subsequent molecular investigation revealed that FER interacts with the transcription factor RESPONSIVE TO DESICCATION 26 (RD26) within the xylem tissue. Given the FER-RD26 interaction and FERONIA's function as a kinase, the authors set out to determine if FER regulates the function of RD26 via phosphorylation. Indeed, in vitro kinase experiments showed phosphorylation of RD26 by FER but not with the kinase-dead *fer* mutant. Furthermore, RD26 protein was more stable in the *fer* mutant compared with the wild type, indicating that FER phosphorylates RD26 to promote its degradation.

These findings begin to paint a picture that RD26 might increase cell wall lignin levels and thereby promote resistance to *R. solanacearum*. To investigate this hypothesis, the authors used an RD26 overexpression line. Soil-drenching experiments with pathogen showed that RD26-overexpression plants significantly delayed wilting symptoms and led to lower pathogen proliferation compared with the wild type and the *rd26* mutant, indicating that RD26 positively regulates plant resistance to bacterial wilt. Transcriptomic studies revealed RD26-mediated enrichment of lignin-related genes, including PAL1. Intriguingly, they found that RD26 binds to the promoter of PAL1 and thereby directly regulates its expression. Together, these findings demonstrate that RD26 plays a positive role in lignin biosynthesis content and enhances resistance to *R. solanacearum* infection.

The FER-RD26 signaling cascade exemplifies a sophisticated molecular mechanism for balancing growth and defense responses. While the RD26 activation enhances pathogen resistance, it compromises growth, as evidenced by the small size of RD26 overexpressing plants. The authors propose that the increased lignin during pathogen infection triggers a feedback loop activating FER, leading to partial RD26 degradation that lowers lignin production. Plants can therefore maintain optimal growth while mounting adequate immune response.

The study uncovers a previously uncharacterized mechanism that regulates lignin accumulation in the vascular system, providing new insights into tissue-specific immune responses. Long term, these findings may inform the development of novel strategies for managing vascular wilt disease and contribute to efforts towards global food security.

## Data Availability

Not applicable.

## Dive Curated Terms

The following phenotypic, genotypic, and functional terms are of significance to the work described in this paper:

PAL1 Gramene: AT2G37040
PAL1 Araport: AT2G37040

FER Gramene: AT3G51550
FER Araport: AT3G51550

RD26 Gramene: At4g27410
RD26 Araport: At4g27410
